# Successful management of pulmonary edema secondary to accidental electrocution in a young dog

**DOI:** 10.1186/s12917-024-03982-4

**Published:** 2024-04-19

**Authors:** Eduardo A. Díaz, Carolina Sáenz, Diana Torres, Andrés Silva, Gilberto Segnini

**Affiliations:** 1 Escuela de Medicina Veterinaria, Colegio de Ciencias de la Salud, Universidad San Francisco de Quito USFQ, Diego de Robles s/n, 170901 Quito, Ecuador; 2 Instituto iBIOTROP, Hospital de Fauna Silvestre Tueri, Universidad San Francisco de Quito USFQ, Diego de Robles s/n, 170901 Quito, Ecuador; 3 Hospital Docente de Especialidades Veterinarias, Universidad San Francisco de Quito USFQ, Diego de Robles s/n, 170901 Quito, Ecuador

**Keywords:** Lung-recruitment maneuver, Mechanical ventilation, Neurogenic pulmonary edema, Point-of-care ultrasound, Vet BLUE

## Abstract

**Background:**

Human records describe pulmonary edema as a life-threatening complication of electric shock. Successful management requires prompt recognition and intensive care. However, in companion animals, electrocutions are rarely reported, even though domestic environments are full of electrical devices and there is always the possibility of accidental injury. Therefore, it is important for veterinarians to know more about this condition in order to achieve successful patient outcomes.

**Case presentation:**

A 3-month-old male Labrador Retriever was presented with a history of transient loss of consciousness after chewing on a household electrical cord. On admission, the puppy showed an orthopneic position with moderate respiratory distress. Supplemental oxygen via nasal catheter was provided, but the patient showed marked worsening of respiratory status. Point-of-care ultrasound exams suggested neurogenic pulmonary edema due to electrical shock close to the central nervous system and increased B-lines without evidence of cardiac abnormalities. Mechanical ventilation of the patient was initiated using volume-controlled mode with a tidal volume of 9 to 15 ml/kg until reaching an end-tidal carbon dioxide ≤ 40 mm Hg, followed by a stepwise lung-recruitment maneuver in pressure-controlled mode with increases of the peak inspiratory pressure (15 to 20 cm H_2_O) and positive end-expiratory pressure (3 to 10 cm H_2_O) for 30 min, and return to volume-controlled mode with a tidal volume of 15 ml/kg until reaching a peripheral oxygen saturation ≥ 96%. Weaning from the ventilator was achieved in six hours, and the patient was discharged two days after admission without neurological or respiratory deficits.

**Conclusions:**

We present a rather unusual case of a neurogenic pulmonary edema subsequent to accidental electrocution in a dog. Timely diagnosis by ultrasound and mechanical ventilation settings are described. Our case highlights that pulmonary edema should be considered a potentially life-threatening complication of electrical shock in small animal emergency and critical care medicine.

## Background

Electric shock injuries are a complex form of trauma often associated with a high rate of morbidity and mortality in veterinary medicine [1]. Most publications describe post-mortem field findings of farm and wild animals due to incidents with high-voltage powerlines or lightning strikes [2–5]. Low-voltage electrocutions in companion animals are rarely reported, even though domestic environments are full of electrical devices and there is always the possibility of accidental injury. This is especially relevant to young dogs, because they are more likely to chew on electrical wires due to their exploratory nature [6].

Human records are more extensive and confirm that the vast majority of electrical shocks in children occur at home due to oral contact with low-voltage electrical wires [7–9]. Low-voltage can cause significant injury to infants who chew wires due to the reduced resistance of moist mucosa and high body water content that allow current to travel more easily [10]. Neurogenic pulmonary edema (NPE) is an underrecognized and underdiagnosed form of pulmonary compromise that can follow central nervous system injury [11]. The associated mortality rate is high, but recovery is usually rapid with appropriate management [12]. NPE secondary to electric shock is a syndrome described in children severe enough to require mechanical ventilation support [13, 14]. Lung-protective mechanical ventilation strategies were associated with decreased mortality in pediatric patients with acute respiratory distress [15].

In veterinary medicine, despite advances in emergencies and critical care, the overall mortality rate for patients undergoing mechanical ventilation remains high, ranging from 61 to 78% [16–18]. Literature includes electrocutions among the possible causes of NPE [19–21], but to the authors´ knowledge, there are no case reports of successful management of NPE with mechanical ventilation after accidental electric shock in small animals. Because pulmonary edema secondary to electrocution can lead to serious complications, even death in young dogs [22, 23], it is important for veterinarians to better characterize the clinical features of this condition. The present study describes the management of a young dog that developed NPE after accidental electrocution, using point-of-care ultrasonography for diagnosis, and lung-protective mechanical ventilation combined with stepwise lung-recruitment maneuver to achieve a successful patient outcome.

## Case presentation

A 3-month-old male Labrador Retriever weighing 9 kg was presented following an accidental electrocution with low-voltage alternating current (110 V). According to the owners, the puppy momentarily lost consciousness after chewing on a household electrical cord. Upon spontaneous recovery, the dog remained agitated and was brought to the hospital within 30 min of the accident.

**Fig. 1:**
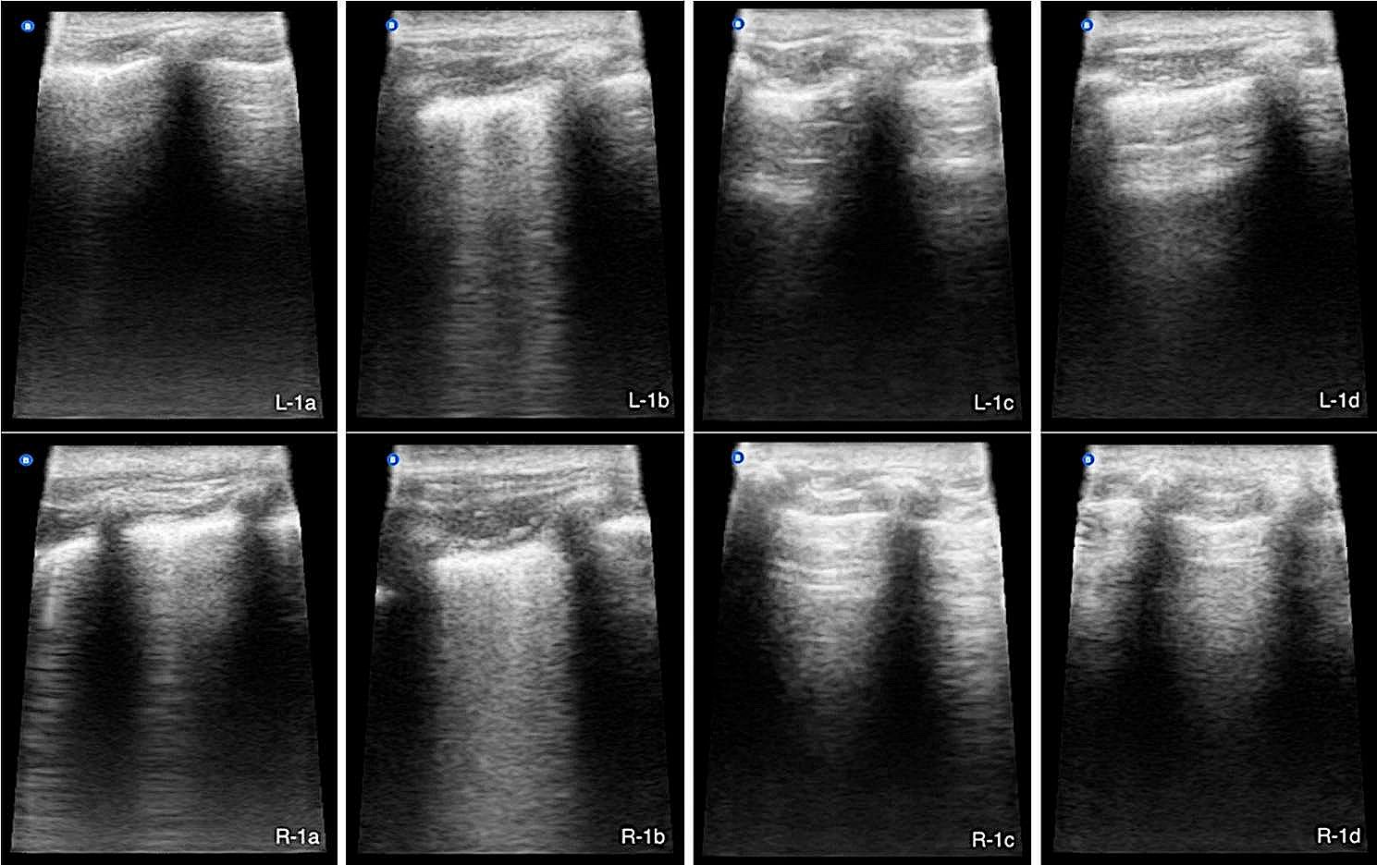
Still B-mode Vet-BLUE images from patient monitoring **1a**: weak positive (≤ 3 B-lines) “wet lung” on admission (L) single B-line in left hemithorax (R) two B-lines in right hemithorax **1b**: strong positive (> 3 B-lines) “wet lung” 30 min post-admission (L) numerous discernible B-lines in left hemithorax (R) numerous indiscernible B-lines in right hemithorax **1c**: negative (0 B-lines) “wet lung” at weaning (L-R) numerous discernible A-lines in both hemithoraces **1d**: “dry lung” 5 days after-discharge (L-R) numerous discernible A-lines in both hemithoraces

On admission, the dog presented in an orthopneic position and with minimal reaction to stimuli. Remarkable physical examination findings included mydriasis, hypersalivation, a grey wound with a surrounding rim of erythema in the mouth (suspected of electrical origin), and moderate respiratory distress characterized by tachypnea (49 bpm), reduced peripheral oxygen saturation (SpO_2_ 92%) and increased end-tidal carbon dioxide (EtCO_2_ 48 mm Hg); the rest of the physiologic parameters were within normal ranges (heart rate 132 rpm, mean arterial pressure 120/80 mm Hg, capillary refill time < 2 s, and rectal temperature 38.5 °C). A veterinary point-of-care ultrasound (V-POCUS) examination, including an abdominal focused assessment with sonography for triage (AFAST), a thoracic focused assessment with sonography for triage (TFAST), and a veterinary bedside lung ultrasound examination (Vet-BLUE), was performed in standing position. The assessment starts from the left side of the patient with the left Vet-BLUE, followed by the left TFAST and then the AFAST. The evaluation is completed on the right side by the right Vet- BLUE and then the right TFAST [24]. The AFAST and TFAST were unremarkable, but the Vet-BLUE showed a weak positive (≤ 3 B-lines) “wet lung” (Fig. 1a). Supplemental oxygen was provided via nasal catheter (2.5 L/min), but after 30 min, the patient worsened and developed cyanosis, increased of respiratory rate (84 bpm), severe hypoxemia (SpO_2_ 80%), and hypercapnia (EtCO_2_ 51 mm Hg) despite oxygen therapy. A second V-POCUS revealed a strong positive (> 3 B-lines) “wet lung” (Fig. 1b), without evidence of cardiac abnormalities, consistent with non-cardiogenic pulmonary edema. Due to the rapidly deteriorating respiratory status, the owners were recommended and accepted mechanical ventilation of the patient.

Anesthesia was induced with propofol (1 mg/kg/min, IV) and maintained using a combination of propofol (0.1 mg/kg/min IV), ketamine (0.3 mg/kg/min IV), and rocuronium (0.2 mg/kg/h IV). The patient was placed in sternal recumbency, and protective mechanical ventilation protocol was started in volume-controlled ventilation mode until EtCO_2_ ≤ 40 mm Hg was reached (Table 1). A stepwise lung-recruitment maneuver was then performed in pressure-controlled ventilation mode for 30 min, with progressive increases of the peak inspiratory pressure (PIP), and positive end-expiratory pressure (PEEP) from 15 to 20 cm H_2_O and 3 to 10 cm H_2_O, respectively (Table 2). Subsequently, the ventilatory mode was returned to volume-controlled ventilation until the SpO_2_ remained ≥ 96% at a fraction of inspired oxygen (FiO_2_) of 50%. Thereafter, the neuromuscular blockade (rocuronium) was discontinued to restore spontaneous breathing, and switched to pressure-support ventilation mode until adequate gas exchange was achieved (SpO_2_/FiO_2_ ≥ 300 mm Hg). At this point, a new ultrasound evaluation revealed a significant pulmonary improvement, with negative (0 B-lines) “wet lung”, and weaning was successfully achieved after six hours of mechanical ventilation (Fig. 1c). The patient recovered smoothly from anesthesia with no respiratory complications.

**Table 1:** Ventilator settings and parameters recorded every 0.5 h during mechanical ventilation of the 3-month-old male Labrador Retriever

Time	VM	TV (ml/kg)	PIP (cm H_2_O)	PEEP (cm H_2_O)	PP (cm H_2_O)	SpO_2_ (%)	FiO_2_ (%)	SpO_2_/FiO_2_ (mm Hg)	EtCO_2_ (mm Hg)	RR (rpm)	HR (bpm)
0	-	-	-	-	-	92	21	438	48	49	132
0.5	-	-	-	-	-	80	30	266	51	84	118
1	VCV	9	18	3	17	99	100	99	50	12	115
1.5	VCV	9	15	4	14	100	100	100	47	12	108
2	VCV	10	15	4	14	99	100	99	44	12	111
2.5	VCV	10	15	4	14	100	100	100	42	12	123
3	VCV	15	15	4	14	100	100	100	40	12	124
3.5	LRM										
4	VCV	15	15	4	14	100	100	100	40	12	134
4.5	VCV	15	15	4	14	100	100	100	45	12	133
5	VCV	15	15	4	14	99	100	99	45	12	142
5.5	VCV	15	15	4	14	99	50	198	42	12	142
6	VCV	14	15	4	14	99	50	198	41	12	124
6.5	PSV	-	8	4	-	99	21	471	40	12	135
7	PSV	-	8	4	-	99	21	471	40	12	144

**EtCO2**, end-tidal carbon dioxide; **FiO2**, fraction of inspired oxygen; **HR**, heart rate; **LRM**, lung-recruitment maneuver; **PCV**, pressure-controlled ventilation; **PEEP**, positive end-expiratory pressure; **PIP**, peak inspiratory pressure; **PP**, plateau pressure; **PSV**, pressure-support ventilation; **RR**, respiratory rate; **SpO2**, peripheral oxygen saturation; **TV**, tidal volume; **VCV**, volume-controlled ventilation; **VM**, ventilatory mode

**Table 2:** Ventilator settings and parameters recorded every 2.5 min during the lung-recruitment maneuver (LRM).

Time	VM	TV (ml/kg)	PIP (cm H_2_O)	PEEP (cm H_2_O)	SpO_2_ (%)	FiO_2_ (%)	SpO_2_/FiO_2_ (mm Hg)	EtCO_2_ (mm Hg)	RR (rpm)	HR (bpm)
2.5	PCV	10	15	3	99	100	99	48	12	124
5	PCV	10	16	3	99	100	99	48	12	124
7.5	PCV	11	17	5	99	100	99	50	12	126
10	PCV	12	18	5	99	100	99	47	12	126
12.5	PCV	13	19	7	99	100	99	47	12	128
15	PCV	14	20	10	100	100	100	42	12	128
17.5	PCV	14	19	10	100	100	100	42	12	128
20	PCV	15	18	7	100	100	100	42	12	136
22.5	PCV	15	17	5	99	100	99	43	12	134
25	PCV	15	16	5	100	100	100	40	12	134
27.5	PCV	15	15	3	100	100	100	40	12	134
30	PCV	15	15	3	100	100	100	40	12	134

**EtCO2**, end-tidal carbon dioxide; **FiO2**, fraction of inspired oxygen; **HR**, heart rate; **LRM**, lung-recruitment maneuver; **PCV**, pressure-controlled ventilation; **PEEP**, positive end-expiratory pressure; **PIP**, peak inspiratory pressure; **PP**, plateau pressure; **PSV**, pressure-support ventilation; **RR**, respiratory rate; **SpO2**, peripheral oxygen saturation; **TV**, tidal volume; **VCV**, volume-controlled ventilation; **VM**, ventilatory mode

Over the next 48 h, the patient was hospitalized in the intensive care unit and maintained with supplemental oxygen via nasal cannula (2.5 L/min), plus a combined IV therapy of furosemide (2 mg/kg q8h), meloxicam (0.2 mg/kg q24h first day then 0.1 mg/kg q24h for an additional day), omeprazole (0.7 mg/kg q24h), N-acetylcysteine (30 mg/kg q8h), and ampicillin/sulbactam (22 mg/kg q8h). Subsequent Vet-BLUEs showed progressive improvement, and discharge was decided 72 h after admission. Home care recommendations included rest and monitoring of neurological or respiratory disorders with N-acetylcysteine (30 mg/Kg q12h PO), plus amoxicillin/clavulanate (22 mg/Kg q12h PO) for five days. No remarkable lung findings were noted on the recheck ultrasound (Fig. 1d), and the owners reported a complete recovery with no sequelae. The dog received no further treatment.

## Discussion and conclusions

To the best of our knowledge, this is the first report of neurogenic pulmonary edema (NPE) associated with low-voltage electrocution in a dog successfully treated with mechanical ventilation. Its uncommon and unpredictable nature, as well as the lack of specific diagnostic markers, may be partly responsible for its low recognition in veterinary medicine. NPE secondary to electric shock is described in human medicine as an underdiagnosed complication of accidental electrocutions [13, 14]. This syndrome has also been reported subsequent to electroconvulsive therapy in children [25]. NPE occurs shortly after a central neurological injury and should be considered when patients suddenly present with respiratory distress. Clinical presentation includes signs of hypoxemia, such as cyanosis, dyspnea, tachypnea, and tachycardia [11]. In veterinary medicine, NPE is described as a possible complication from a variety of brain injuries, including electrocution, requiring prompt recognition due to rapid deterioration of respiratory status [20]. Diagnosis is generally based on history, clinical signs, diagnostic imaging findings, and exclusion of other causes of pulmonary edema [21]. V-POCUS exams include non-invasive, radiation-sparing, and cost-effective monitoring techniques that enable rapid assessment of respiratory failure, improving decision-making in the emergency service [26, 27]. Specifically, the Vet-BLUE scoring system helps in classifying serial point-of-care lung images into clinical information. The development of pulmonary edema is correlated to the increase in B-lines [28]. In the present case, there was no history to suggest any previous pulmonary or cardiac disease, so given the altered state of consciousness following the electrical shock close to the central nervous system, the rapid onset of respiratory signs, and the increased B-lines without evidence of cardiac failure, the possibility of NPE was considered likely.

Although NPE is described as life-threatening in companion animals, no specific therapies have been developed for this condition, and the mainstay of treatment is based on removal of the inciting cause and supportive care [20, 21]. More specific human protocols include lung-protective mechanical ventilation to improve hypoxemia secondary to NPE without additional lung injury. However, protective mechanical ventilation may be challenging due to the requirement of low tidal volume ventilation and permissive hypercapnia that could worsen clinical sings [11]. Dogs requiring lung-protective mechanical ventilation due to pulmonary pathologies traditionally received lower tidal volume than healthy dogs, but the tidal volume may be broader than what is generally recommended for lung-protective strategies, ranging from 10.15 to 14.96 ml/kg. The differences between humans and dogs may be due to a greater basal metabolism and physiologic dead space of dogs, which may explain a greater metabolic production of CO2, and justify the use of a higher tidal volume to avoid excessive accumulation of CO2 [29, 30].

Early administration of neuromuscular blocking agents has been used in human medicine to facilitate endotracheal intubation, prevent ventilation asynchrony, improve oxygenation, decrease barotrauma, and reduced the duration of mechanical ventilation and mortality [31]. The combination of neuromuscular blocking agents, with prone positioning of mechanically ventilated patients, may exert a synergistic protective effect on the lungs [32]. In addition, there is evidence supporting the use of stepwise increases in positive end-expiratory pressure, with the goal of mitigating the prolonged high pulmonary pressure used in sustained inflation and increasing the recruitment time in human patients with acute respiratory distress [33, 34]. The use of neuromuscular blocking agents has also been described in strategies for mechanical ventilation of small animals [35, 36]. Experimental studies have shown that lung-protective ventilation in sternal recumbency, combined with the recruitment maneuver, improves oxygenation while reducing the risk of ventilator-induced lung injury in dogs with acute respiratory stress [37, 38]. A stepwise recruitment maneuver rather than sustained inflation is also recommended in veterinary literature. Once the recruitment maneuver is complete, the positive end-expiratory pressure should be adjusted to prevent de-recruitment [35, 39]. Therefore, it was decided to perform neuromuscular blockade, in combination with the sternal recumbency positioning of the patient and the incorporation of the stepwise recruitment maneuver, during mechanical ventilation.

Therapies for NPE in humans also include control of circulatory volume with diuretics for resolution of pulmonary edema [11]. However, volume management balance is not always easy, because the low circulating volume that can reduce pulmonary edema could cause cerebral hypoperfusion. Real-time ultrasound provides an accurate assessment of pulmonary interstitial fluid that can guide on volume management [40, 41]. The use of diuretics for pulmonary edema in small animals is controversial. Furosemide is recommended for the treatment of cardiogenic pulmonary edema in which preload and left atrial pressure are increased. These parameters are not altered in non-cardiogenic pulmonary edema and, although furosemide may play a role in reducing pulmonary capillary pressures, the transient nature of its causes makes it unlikely to be helpful [42, 43]. Although NPE has traditionally been described as a non-cardiogenic form of pulmonary edema, there is evidence in human patients that neurological damage can lead to myocardial injury and the development of pulmonary edema [11, 44]. The Vet-BLUE can rapidly detect signs of pulmonary edema but cannot provide a definitive diagnosis for underlying cause of lung pathology [45]. Thus, given the impossibility of completely ruling out an overlap of neurogenic and cardiogenic pulmonary edema, treatment with furosemide was decided. We use the Vet-BLUE as a guide for diuretic therapy by monitoring resolution of B-lines.

Published veterinary management for oral electrical burns is sparse, but conservative approaches recommend prophylactic antibiotic treatment in human medicine, particularly when patients chew alternating current cords, due to possible necrosis of affected tissue caused by prolonged exposure to the electrical source resulting from tetanic contraction of the masticatory muscles [46]. Although systemic antibiotic prophylaxis is not always recommended in the treatment of burns, it may be useful in patients who require mechanical ventilation due to the risk of pneumonia [47]. Furthermore, while ventilator-associated pneumonia is one of the most common nosocomial infections, there is evidence in human medicine that the early use of antibiotic prophylaxis may prevent its occurrence in intensive care patients [48–50]. Infections associated with mechanical ventilation have also been described in small animals and, although further studies are needed to evaluate the effect of antimicrobial therapy on patient outcome [51, 52], we considered the use of prophylactic antimicrobial therapy reasonable, given that the puppy had an oral electrical burn and required mechanical ventilation.

In the veterinary literature, there are only two previous reports of presumed neurogenic pulmonary edema in dogs associated with accidental electrocution. Yamamoto [22] describe the case of a 6-month-old Beagle and a 3-month-old Yorkshire with excessive salivation, prostration, intense dyspnea, and labored breathing. In both cases, radiographs showed a diffuse alveolar pattern of pulmonary edema, without alteration in the cardiac silhouette. Oxygen therapy, as well as dexamethasone, aminophylline, furosemide, amoxicillin, and analgesics, were administered. The beagle had a progressive improvement and after 48 h was discharged. The Yorkshire did not respond well to the treatment and death happened after 12 h. Singh [23] presents the case of a 40-days-old German Shepherd with severe respiratory distress and convulsions. X-rays revealed broncho-interstitial pneumonia without alteration in the cardiac silhouette. Treatment involved oxygen therapy along with parenteral corticosteroids, fluid, antihistaminic, antibiotics, diuretics, and B-complex administration. The puppy succumbed 12 h after initiation of treatment. Although traditionally thoracic radiography has been considered a diagnostic test in small animals, non-cardiogenic pulmonary edema may present with a rather variable radiographic appearance, complicating its diagnosis [53]. Furthermore, the main goal of its therapy is to preserve tissue oxygenation. This may be achieved by supplemental oxygen in mild-moderate cases but require mechanical ventilation in patients with severe respiratory distress [21]. It is likely that the high mortality evidenced in these case reports is due to the lack of an accurate diagnosis and the need for mechanical ventilation of patients.

This study has several limitations. First, furosemide can cause decrease in tissue perfusion that worsens the patient´s clinical signs, so its use for the treatment of possible cardiogenic edema should be corroborated by echocardiographic parameters and serum cardiac biomarkers [54]. Second, neuromuscular blockade benefits must be weighed against the possible adverse effects, and the knowledge gaps about its use in small animals and the risks should be reduced with neuromuscular monitoring and the use of reversal blocking agents [55]. Third, inappropriate use of antimicrobials can lead to resistance, and antibiotic therapy should have been based on bacterial cultures and resistance patterns to reduce unnecessary and inappropriate use of antimicrobials [56]. Lastly, the direct application of available data from human to veterinary medicine is not always advisable, therefore, further research is needed to increase the reliability of diagnostic tests and treatments, considering the intrinsic pathophysiology of pulmonary edema in small animals [35]. Unfortunately, the patient´s life-threatening situation and owner´s financial constraints did not allow for additional diagnostic testing in the present case.

In conclusion, pulmonary edema is an uncommon but potentially life-threatening complication of accidental electric shock in dogs. The history, clinical signs, and diagnostic imaging findings suggest neurologically mediated pulmonary edema. NPE should be considered in patients with rapid deterioration of respiratory status after electrocution-induced central nervous system insults. It is important that the critical care veterinarian is familiar with the management of this condition in order to improve decision-making in the emergency service. In addition, pet owners should be informed during routine visits to the veterinarian about the importance of preventing electrical accidents in the domestic environment, especially in puppies and kittens.

## Data Availability

The data presented in this study would be available on request from the corresponding author.

## Abbreviations

EtCO2: End-tidal carbon dioxide
FAST: Focused assessment sonography for trauma
FiO2: Fraction of inspired oxygen
LRM: Lung-recruitment maneuver
NPE: Neurogenic pulmonary edema
PEEP: Positive end-expiratory pressure
PIP: Peak inspiratory pressure
PP: Plateau pressure
SpO2: Peripheral oxygen saturation
TV: Tidal volume
Vet-BLUE: Veterinary bedside lung ultrasonography examination
*V-POCUS*: *Veterinary point-of-care ultrasound*
